# Influence of Metal‐Alkyls on Early‐Stage Ethylene Polymerization over a Cr/SiO_2_ Phillips Catalyst: A Bulk Characterization and X‐ray Chemical Imaging Study

**DOI:** 10.1002/chem.202002632

**Published:** 2020-12-09

**Authors:** Maarten K. Jongkind, Florian Meirer, Koen W. Bossers, Iris C. ten Have, Hendrik Ohldag, Benjamin Watts, Theo van Kessel, Nic. Friederichs, Bert M. Weckhuysen

**Affiliations:** ^1^ Inorganic Chemistry and Catalysis Debye Institute for Nanomaterial Science Utrecht University Universiteitsweg 99 3584 CG Utrecht The Netherlands; ^2^ Technology and Innovation Department SABIC Urmonderbaan 22 6167 RD Geleen The Netherlands; ^3^ Advanced Light Source, Microscopy Lawrence Berkeley National Laboratory 1 Cyclotron Road Berkeley CA 94720 USA; ^4^ Department of Materials Science and Engineering Stanford University 450 Serra Mall Stanford CA 943505 USA; ^5^ Department of Physics University of California Santa Cruz 1156 High Street Santa Cruz CA 95064 USA; ^6^ Laboratory for Synchotron Radiation—Condensed Matter (LSC) Paul Scherrer Institute (PSI) Forschungsstrasse 111 5232 Villigen Switzerland

**Keywords:** chromium, Phillips catalyst, polyethylene crystallinity, polymerization catalysis, scanning transmission X-ray microscopy

## Abstract

The Cr/SiO_2_ Phillips catalyst has taken a central role in ethylene polymerization since its invention in 1953. The uniqueness of this catalyst is related to its ability to produce broad molecular weight distribution (MWD) PE materials as well as that no co‐catalysts are required to attain activity. Nonetheless, co‐catalysts in the form of metal‐alkyls can be added for scavenging poisons, enhancing catalyst activity, reducing the induction period, and tailoring polymer characteristics. The activation mechanism and related polymerization mechanism remain elusive, despite extensive industrial and academic research. Here, we show that by varying the type and amount of metal‐alkyl co‐catalyst, we can tailor polymer properties around a single Cr/SiO_2_ Phillips catalyst formulation. Furthermore, we show that these different polymer properties exist in the early stages of polymerization. We have used conventional polymer characterization techniques, such as size exclusion chromatography (SEC) and ^13^C NMR, for studying the metal‐alkyl co‐catalyst effect on short‐chain branching (SCB), long‐chain branching (LCB) and molecular weight distribution (MWD) at the bulk scale. In addition, scanning transmission X‐ray microscopy (STXM) was used as a synchrotron technique to study the PE formation in the early stages: allowing us to investigate the produced type of early‐stage PE within one particle cross‐section with high energy resolution and nanometer scale spatial resolution.

## Graphical abstract

**Tailoring polymerization processes**: The addition of the proper type and amount of co‐catalyst in a Cr/SiO_2_ Phillips‐type ethylene polymerization system tailors the molecular weight distribution and degree of short chain branching. We have now used scanning transmission X‐ray microscopy for investigating how these properties affect the polyethylene crystallinity in the early stages of ethylene polymerization.

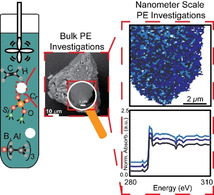

## Introduction

The production of polyethylene (PE) is estimated to increase to an annual production of over 100 million tons in 2020 and continues to be a ubiquitous material in our society in the decades to come.^[1]^ In the catalytic production of PE three main pillars can be identified, namely Ziegler–Natta catalysts,[^2^, ^3^] (post‐) metallocenes,^[4]^ and Phillips‐type catalysts.[^1^, ^5^, ^6^, ^7^, ^8^, ^9^, ^10^]

Ever since its invention by Hogan and Banks at the Phillips Petroleum Company laboratories in 1953,[^11^, ^12^] the Cr/SiO_2_ Phillips catalyst has evolved to such an extent that nowadays it is responsible for approximately 30 % of all high‐density polyethylene (HDPE) manufactured world‐wide. Due to the extensive industrial usage of the Cr/SiO_2_ Phillips catalyst it has received a lot of interest from both industrial and academic research. However, no consensus has been reached yet on the activation mechanism of this catalyst, and the related ethylene polymerization mechanism.

The Cr/SiO_2_ Phillips catalyst is unique in comparison to the other two ethylene polymerization catalysts: while Ziegler–Natta and Group 4 transition metal based (post‐) metallocene type catalysts require the addition of a metal‐alkyl as co‐catalyst for activation, these compounds are not a necessity for the activation of Phillips‐type ethylene polymerization catalysts. Here, ethylene can fulfil the dual role of activator and monomer source. However, metal‐alkyls, often in the form of alkyl‐boron or alkyl‐aluminum compounds, can be added to scavenge poisons, reduce the induction period, enhance catalyst activity and tailor polyethylene product properties.^[1]^

A complete understanding of the activation mechanism and related ethylene polymerization mechanism are hindered by at least two reasons. Firstly, weight‐loadings higher than 1 wt % are avoided due to a decrease in catalytic activity related to the formation of Cr clusters, or at least oligomeric species. Secondly, only a portion, proposedly a maximum of 30 %,^[1]^ of the Cr sites is active in ethylene polymerization. These two key‐points made necessary the use of various powerful analytical approaches, often avoiding industrial conditions and/or catalyst materials by use of, for example, model systems[^13^, ^14^, ^15^, ^16^, ^17^, ^18^, ^19^, ^20^, ^21^, ^22^, ^23^] or well‐defined catalysts in which Cr^6+^ is reduced by for example, CO: both examples limiting the heterogeneities of the surface sites, in the former case by rationally designing the Cr surface sites and in the latter by quantitative reduction to isolated Cr^2+^ surface sites.[^24^, ^25^, ^26^, ^27^, ^28^, ^29^, ^30^, ^31^, ^32^]

One example of a long‐standing discussion is the oxidation state of the Cr active site, which has been investigated with many different spectroscopic techniques, demonstrating that the active valency lies between 2 and 3, with the true value still being questioned by varying insights from different research groups. These differences, however, can in part be ascribed to different reaction set‐ups and different catalyst materials.[^33^, ^34^, ^35^, ^36^, ^37^, ^38^, ^39^, ^40^, ^41^, ^42^, ^43^, ^44^, ^45^, ^46^]

Another extraordinary aspect, and long‐standing point of discussion, is the large variety of PEs that can be produced with the Phillips catalyst and the multitude of strategies, such as calcination temperature, support type, co‐catalyst addition and catalyst pre‐treatment, one can employ in tailoring the final PE product properties.[^43^, ^47^, ^48^, ^49^, ^50^, ^51^, ^52^, ^53^, ^54^, ^55^] The diversity of produced PEs, in terms of PE‐type as well as the broad molecular weight distributions (MWD), is ascribed to the large variety of Cr surface sites. Each surface site produces an average type of PE,[^56^, ^57^, ^58^] in which the active sites are not regarded as naked Cr ions: instead, reduction by‐products remain important constituents of the active sites during polymerization.[^27^, ^44^, ^57^, ^59^, ^60^, ^61^] For example, PE properties produced from an AlPO_4_‐supported Cr catalyst can be controlled by the precise addition of tri‐ethyl borane (TEB) to the polymerization process, whereas the SiO_2_ analogues were less sensitive to this metal‐alkyl.[^34^, ^43^, ^51^, ^62^, ^63^, ^64^] Despite the widespread industrial usage of metal‐alkyl co‐catalysts, such as TEB and tri‐ethyl aluminum (TEAl), owing to their ability to increase control over PE product properties, they have only started to receive increased academic interest in recent years.[^56^, ^57^, ^59^, ^65^, ^66^, ^67^]

One example is a study from our group, in which the influence of TEAl on the polymerization properties of a shell‐titanated Phillips catalyst (i.e. Cr/Ti/SiO_2_) was investigated and demonstrated how the addition of TEAl as co‐catalyst increased the oligomerization of ethylene. Scanning transmission X‐ray microscopy (STXM) was used to reveal that within one catalyst particle the titanium‐rich shell‐region produced a linear PE, whereas the titanium‐poor center‐region of the catalyst particle generated a linear low density polyethylene (LLDPE), demonstrating how different PE materials can be distinguished at the nanometer‐scale.[^68^, ^69^, ^70^]

Here, we present a study in which STXM offered the possibility to investigate the type of early‐stage PE produced by a Cr/SiO_2_ Phillips catalyst as a function of different amounts of TEB and TEAl.^[71]^ In addition, semi‐batch ethylene polymerization reactions at constant polymer yields allowed us to study catalyst activities as well as the resulting polymers, which were studied in terms of molecular weight (*M*_w_), molecular weight distribution (MWD, *M*_w_/*M*_n_), short‐chain branching (SCB) and long‐chain branching (LCB) by bulk characterization techniques, namely size exclusion chromatography (SEC) and ^13^C NMR.[^72^, ^73^] These data allowed us to correlate the type of PE produced at the level of a single catalyst particle cross‐section with that of bulk PE.

## Results and Discussion

For this work, we have selected a Cr/SiO_2_ Phillips catalyst, consisting of 1 wt % Cr impregnated on a 625 m^2^ g^−1^ SiO_2_ porous support, activated by a proprietary calcination method at 650 °C. The ethylene polymerization performance of this catalyst in the presence of small amounts of TEB and TEAl as co‐catalyst was investigated by means of a 5 L semi‐batch reactor, in which the amount of co‐catalyst was present in ppm (wt/wt) levels. The produced PE materials were subsequently characterized with SEC‐DV‐IR and ^13^C NMR.

In a second stage of our study, at key co‐catalyst mole ratios of, respectively 1.5 and 5.0 m:Cr (M=B/Al) of TEB and TEAl, the ethylene polymerization reactions over the Cr/SiO_2_ Phillips catalyst were quenched before the onset of catalyst particle fragmentation. The produced catalyst‐PE materials were cut into slices of about 100 nm by ultra‐microtomy, after embedding in a hard immobilizing epoxy resin, and scanning transmission X‐ray microscopy (STXM) was used to study the type of PE produced while it was still largely dispersed within the Cr/SiO_2_ catalyst phase, with polymer yields between 1–2 g_PE_ g_cat_^−1^.

In the following we will first present the ethylene polymerization results, and the related characterization of the polyethylene produced by a Cr/SiO_2_ Phillips catalyst as a function of the amount of TEB and TEAl. In the second part, we will focus on the STXM data of early‐stage ethylene polymerization Cr/SiO_2_ Phillips catalyst materials, which, in a final third part, will be compared to reference materials and rationalized with the bulk PE properties.

### Effect of metal‐alkyls on the bulk polyethylene properties

**Figure 1:**
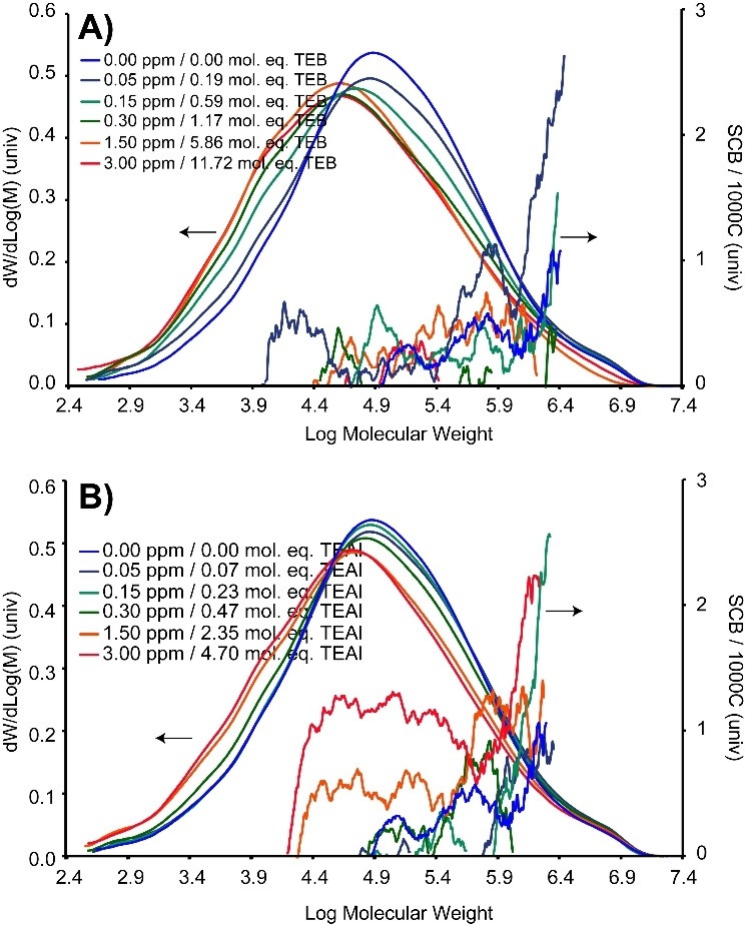
Results obtained from size exclusion chromatography*–*differential viscometry*–*infrared (SEC‐DV‐IR) measurements on the produced polymers with different amounts of A) tri‐ethyl borane (TEB) and B) tri‐ethyl aluminum (TEAl), demonstrating the molecular weight distribution (MWD; arrow pointing left) as well as the degree and distribution of short chain branching (SCB; arrow pointing right).

Two properties that govern a significant portion of the final PE characteristics are the molecular weight distribution (MWD) as well as branching, consisting of short‐chain branching (SCB) and long‐chain branching (LCB),[^52^, ^55^, ^74^] the latter being defined as a sidechain with over 150 carbon atoms. Table 1 illustrates how, respectively TEB and TEAl affect the mass average molar mass (*M*_w_), the number average molar mass (*M*_n_), the *z*‐average molar mass (*M*_z_) and the poly‐dispersity index (PDI, *M*_w_/*M*_n_). TEB decreases the M_n_ after the addition of only 0.19 mol. eq. TEB (0.05 ppm) to 15 KDa and further to a minimum of 9 KDa in the presence of 11.72 mol. eq. TEB (3.00 ppm). The relative increase of shorter PE molecules is further corroborated by an increasing MWD, as demonstrated in Figure 1 A. The *M*_w_ and *M*_z_ values reveal that the broadening MWD is mostly caused by a relative larger number of low MW PE chains, since both the *M*_w_ and *M*_z_ take chain‐length into account, with a stronger contribution of the larger PE molecules to the final value.^[74]^ Raising the amount of TEB to 5.86 mol. eq. (1.50 ppm) and above results in a significant decrease of the *M*_w_ and *M*_z_, indicating that from hereon also a relatively smaller number of the higher MW PEs is observed, further corroborated, in Figure 1 A.

**Table 1:** Molecular structure parameters obtained from the size exclusion chromatography–differential viscometry–infrared (SEC‐DV‐IR) analysis of the polyethylene (PE) produced in a 5 L semi‐batch reactor during ethylene polymerization over a Cr/SiO_2_ catalyst for various amounts of tri‐ethyl borane (TEB) and tri‐ethyl aluminum (TEAl) as co‐catalysts.

Sample	M:Cr mole ratio (M=B/Al)	*M*_n_ [kDa]	*M*_w_ [kDa]	*M*_z_ [kDa]	*M*_w_/*M*_n_	Density [kg m^−3^]	Averaged catalyst activity [kg_P*E*_ kg_cat_^−1^ min^−1^]
0.00 ppm	0	19	360	2600	18.6	956.1	14.8
**TEB**							
0.05 ppm	0.19	15	380	2900	26.2	957.8	44.4
0.15 ppm	0.59	13	350	2900	25.9	959.4	52.8
0.30 ppm	1.17	11	330	3000	30.3	960.0	90.9
1.50 ppm	5.86	11	220	1700	20.8	958.8	69.9
3.00 ppm	11.72	9	270	2400	29.1	958.9	59.3
**TEAl**							
0.05 ppm	0.08	18	400	2900	22.1	956.4	30.5
0.15 ppm	0.23	19	370	2600	19.8	956.3	39.6
0.30 ppm	0.47	16	370	2900	23.6	954.6	42.2
1.50 ppm	2.35	12	340	2900	29.3	953.4	52.9
3.00 ppm	4.70	12	320	2900	28.0	953.5	50.5

TEAl affects the MWD to a smaller extent. Indeed, Table 1 shows a constant *M*_n_ up until 0.47 mol. eq. TEAl (0.30 ppm, which is in line with the constant MWD, as demonstrated in Figure 1 B. After raising the co‐catalyst amount the MWD increases, however, unlike for TEB, the PE fraction in the log(MWD) range of 6.4–6.9 remains constant, also testified by the constant *M*_z_. Furthermore, the relatively constant MW up until a TEAl value of 2.35 mol. eq. (1.50 ppm), indicates that TEAl mostly affects the relative amount of lower MW PE.

**Figure 2:**
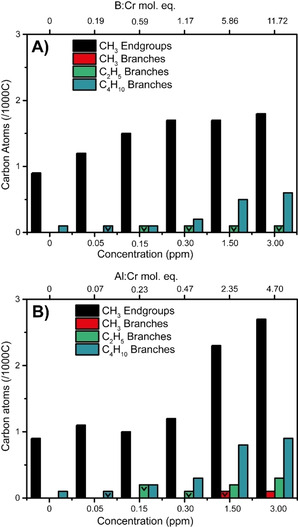
End‐group analysis of the polymers produced with A) tri‐ethyl borane (TEB) and B) tri‐ethyl aluminum (TEAl) measured with ^13^C NMR giving the number of methyl, ethyl, and butyl short‐chain branches (SCB) as well as methyl end‐groups.

The presence of TEB or TEAl also uniquely affects the degree of SCB, as is demonstrated in Figures 1 and Figure 2, with the triangles in Figure 2 reflecting values „smaller than“. Firstly, it was found that the presence of TEAl is directly correlated to enhanced SCB, and, that this is predominantly observed for the PE produced in the presence of 2.35 and 4.70 mol. eq. TEAl (respectively 1.50 ppm and 3.00 ppm). All of the short‐chain branches reside on the longer chains if TEAl amounts up to 0.47 mol. eq. (0.30 ppm) were used. However, at values of 2.35 mol. eq. (1.50 ppm) and 4.70 (3.0 ppm) mol. eq. they also reside on the smaller PE chains. Enhanced α‐oligomer generation and incorporation is the underlying reason for this observation.

On the other hand, the presence of TEB has a less pronounced effect on the SCB. At TEB molecular equivalencies of 0.59 (0.15 ppm) and higher only minor amounts of butyl branches are detected and ethyl branches are detected even less. Interestingly, increasing the amount of TEB to 5.86 mol. eq. (1.50 ppm) and higher resulted in a small increase of the butyl branches, however there was no noticeable effect on the amount of ethyl branches.

Furthermore, the densities presented in Table 1 are not just an effect of SCB and to a lesser extent by the *M*_w_, where the densities of the materials produced with TEB are higher than those produced with TEAl (Figure S8). Likely, this caused by the lower Mn as well as the SCB distribution for the materials produced with TEB, with the SCB distribution demonstrating that this does not really occur on the smaller MW PE chains, in turn resulting in high crystalline PE fraction. On the other hand, for TEAl there is initially little to no broadening of the MWD, thus these crystalline domains are absent: resulting in overall lower densities of these PEs. In case the MWD does broaden, with amounts of 2.35 mol eq. and 4.70 mol. eq. TEAl (respectively 1.50 and 3.00 ppm), SCB is also observed at lower MWD chains, thus prohibiting the formation of high‐crystalline domains.

**Figure 3:**
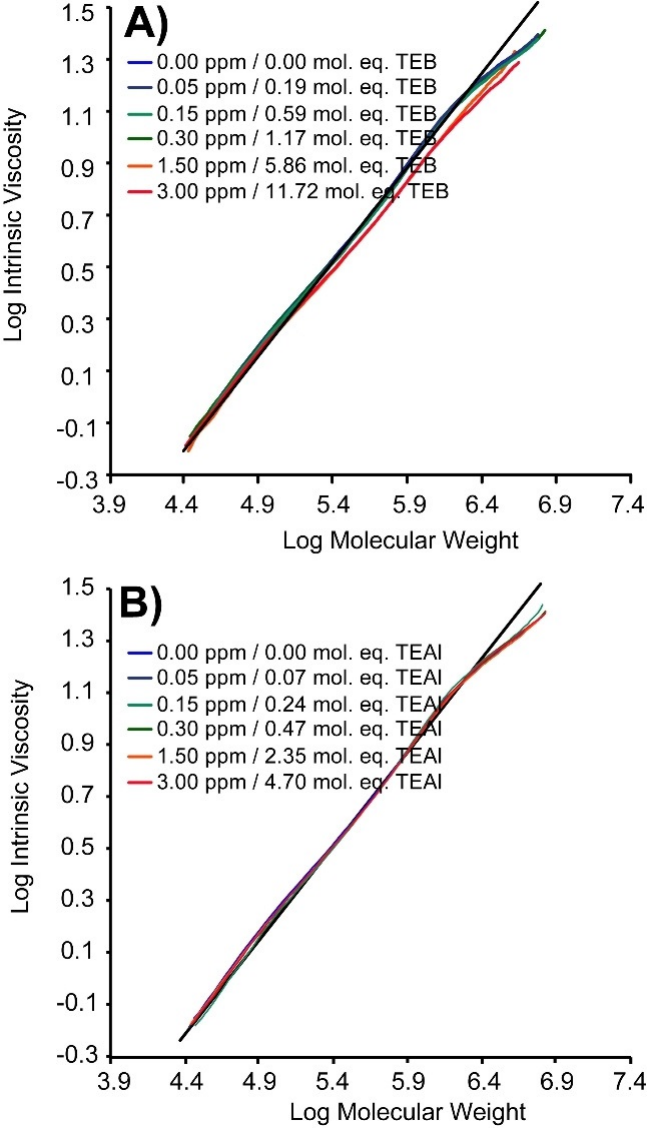
Mark–Houwink plots obtained from the size exclusion*–*differential viscometry*–*infrared (SEC‐DV‐IR) measurements for the polyethylene materials produced with A) TEB and B) TEAl, in which the intrinsic viscosity of the produced PE materials was compared to those of linear PE materials. PE molar mass calibration was performed with linear PE standards in the range of 0.5*–*2800 kg mol^−1^ (*M*_w_/*M*_n_=4 to 15).

Both co‐catalysts also have some effect on the presence, or absence, of LCB,[^75^, ^76^] as shown in Figure 3, none of the materials perfectly fit the theoretical correlation for linear PE, however it is only with increased amounts of TEB, 5.86 mol. eq. (1.50 ppm) and above, that the PEs deviate more significantly from the linear reference line.

If the *M*_n_ is used to calculate the theoretical number of CH_3_ end‐groups per 1000 C atoms, as illustrated in Figure S**7**, we see that in the case of 11.86 mol. eq. TEB (3.00 ppm), ≈1.8 CH_3_ end‐groups per 1000 C are observed, which is close to the theoretical value of 1.55, under the assumption of 1 CH_3_ end‐group per PE chain. TEAl on the other hand, with an *M*_n_ of 12 should have a theoretical amount of ≈1.2 CH_3_ end‐group in the absence of LCB, however the measured amount is closer to 2.75 with 4.70 mol. eq. (3.00 ppm). This value on its own might be interpreted as LCB, however, the Mark–Houwink plot in Figure 3 demonstrates only minor deviations from the linear references. In the case of TEB, an amount close to 1 CH_3_ end‐group per polymeric chain is observed. What is exactly responsible for this increased number of CH_3_ end‐groups at relatively high amounts of TEAl remains elusive, but since TEAl is now present in excess, it might act now as a chain transfer agent as well. Possibly other elimination mechanisms from the aluminum‐polymer complex proliferate, resulting in 2 CH_3_ end‐groups per chain; however, this remains speculation and requires further investigation.

### Scanning transmission X‐ray microscopy of polyethylene reference materials

**Figure 4:**
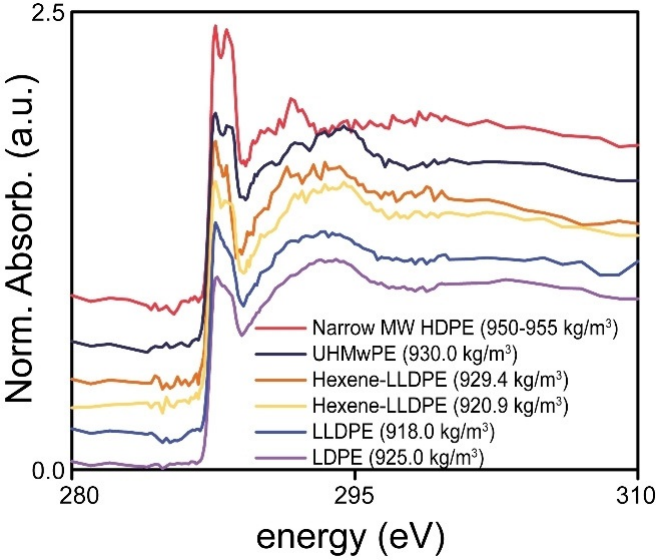
The results of the scanning X‐ray transmission microscopy measurements (STXM) on the reference materials on the C K‐edge.

One of the key‐challenges is to compare the produced early‐stage materials to bulk polymers. For this, a serie of reference materials was investigated with STXM, their bulk C K‐edge XANES, shown in Figure 4. These reference materials were rationally selected to cover a large variety of PE materials in terms of density.

Figure 4 illustrates the findings by Schöll et al.^[77]^ who found that PE density, and related crystallinity, coincides with the line shape of the 287.4 and 287.8 eV σ*_C−H_ transitions. Most importantly, Figure 4 shows that for a narrow MW HDPE, with a density of 950–955 kg m^−3^, the splitting of this signal is apparent and the signals can clearly be identified. The trend that emerges from Figure 4 is that upon decreasing the density, the splitting of the 287.4 and 287.8 eV σ*_C−H_ transitions becomes more obscured, which also holds for the σ*_C−C_ transitions. One exception is, however, the LDPE (925 kg m^−3^) reference material, for which the density was higher than two of the LLDPE (918 kg m3, 920.9 kg m^−3^) references, nonetheless, the splitting was more obscure: this is likely due to LCB in this material, which was absent in all other instances.

### Scanning transmission X‐ray microscopy of the early‐stage ethylene polymerization over Cr/SiO_2_ catalysts

The analysis of the bulk polymers has shown that it is possible to tailor the PE properties, in terms of predominantly MWD and SCB, with the proper selection of the type and amount of co‐catalyst. However, it is still unclear how these differences affect early‐stage PE materials. The fact that the C K‐edge XANES line shape is directly related to the PE density allows us to correlate the type of early‐stage PE to bulk PE references. STXM offers the opportunity to generate high spatial‐resolution image‐sequences at specified high‐resolution X‐ray energies (Δ*E*≈0.1 eV at edge‐jump), allowing for the spatial correlation of different PE densities at the nanometer scale via their characteristic so‐called X‐ray Absorption Near Edge Structure (XANES). However, individual PE crystallite domains (≈27 nm) are below our used spatial resolution (100 nm^2^), as a consequence, each pixel is (possibly) a sum of multiple randomly oriented PE crystallites.^[78]^ Improving the STXM technology further is technically possible but also limited by the spectral signal to noise ratio when inspecting such small volumes containing tiny amounts of polymer, as is the case for these early‐stage materials. To complement the spatially resolved STXM measurements, DSC was used as a tool to quantify the melting temperatures (T_m_) of these materials.

It is worth mentioning that linear dichroism can occur for crystalline PE materials in STXM measurements. However, this often requires highly crystalline samples prepared with special methods and on special (poly‐)crystalline substrates. The requirement of highly‐ordered, preferably single‐crystalline samples, is not fulfilled by our materials, since they show 1) broad MWD, 2) are grown randomly over 3) a large variety of active sites, leading to azimuthally averaged distribution of early‐stage PE materials and 4) are not grown on (poly‐)crystalline substrates.^[79]^

We used equimolar amounts of TEB or TEAl in the preparation of the early‐stage materials, to effectively compare their effects on the type of early‐stage PE, and selected key amounts of 1.5 and 5.0 mol. eq. (M: Cr; M=B/Al). Firstly, 1.5 mol. eq. of TEB is expected to result in a broadened MWD, however should demonstrate very little SCB. By increasing the amount of TEB to 5.0 mol. eq., the expected degree of SCB was enhanced and the MWD is expected to broaden. Secondly, a comparison of the PE produced with 1.50 mol. eq. of TEAl shows, based on Figure 1, that almost no broadening of the MWD is expected, whereas the degree of SCB is expected to be larger. By raising the amount of TEAl to 5.0 mol. eq., the MWD is expected to broaden, whereas the degree of SCB is largely retained. In summary, we have 4 samples with the following characteristics: **1)** very small amounts of SCB (1.5 mol. eq. TEB), **2)** increased SCB (1.5 mol. eq. TEAl), **3)** significantly broadened MWD and small amount of SCB (5.0 mol. eq. TEB) and **4)** broadened MWD with increased SCB (5 mol. eq. TEAl). An extended overview of the sample preparation is given in the experimental section and the Supporting Information.

**Figure 5:**
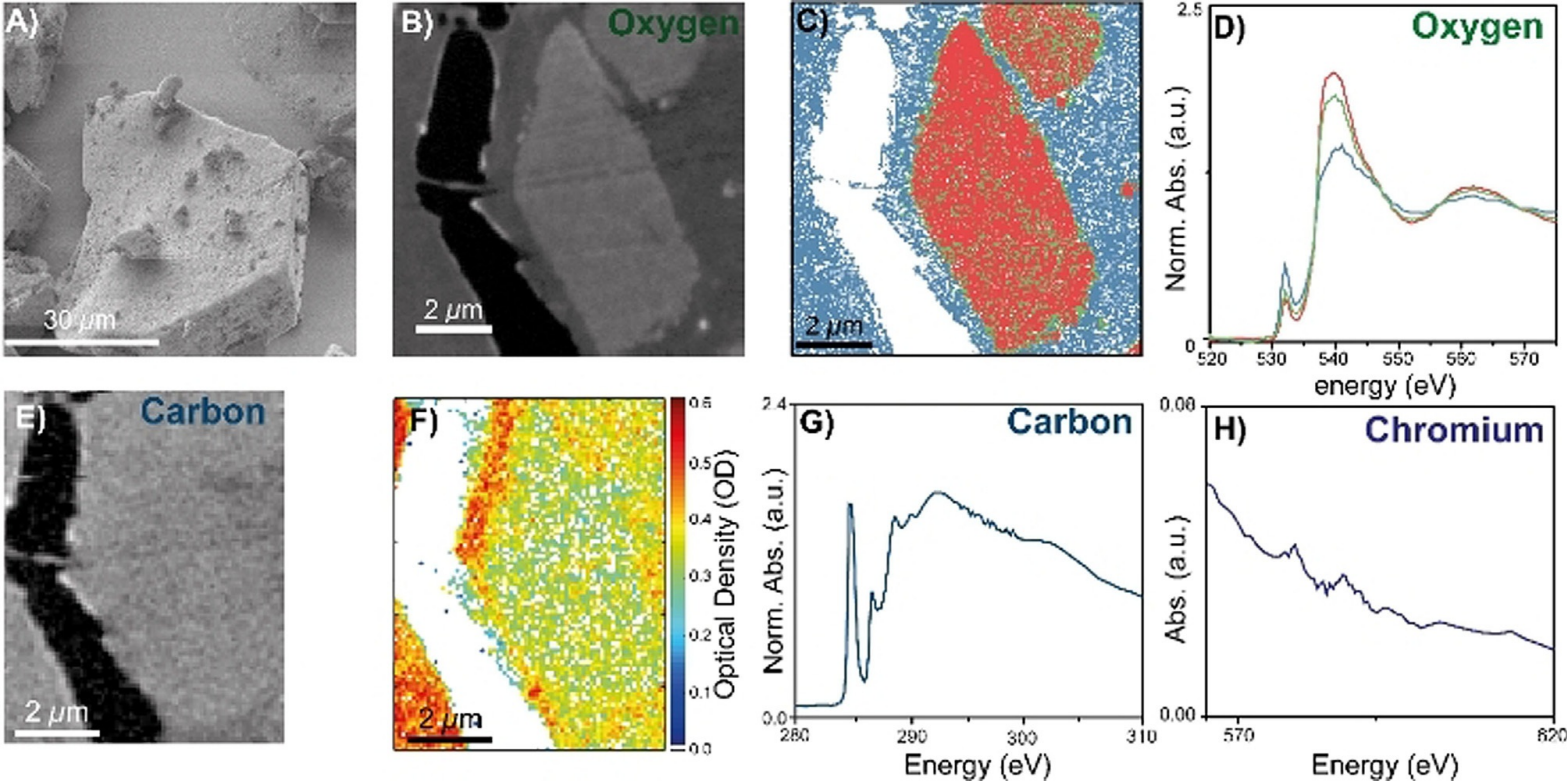
A schematic overview of the performed scanning transmission X‐ray microscopy (STXM) measurements on the Cr/SiO_2_ Pristine catalyst. A) Scanning electron microscopy (SEM) image of the pristine catalyst. B) Optical density (OD) image at the O K‐edge (538 eV) of the microtomed cross‐section of the pristine catalyst. C) Clustered image after principal component analysis (PCA) at the O K‐edge, showing three phases: oxygen from the Struers Epofix epoxy resin and two mixed phases. D) O K‐edge X‐ray absorption near‐edge spectra (XANES) prior to the linear subtraction of the epoxy reference O K‐edge XANES from the combination spectra. E) STXM OD image at the C K‐edge (280 eV) of the microtomed cross‐section. F) Carbon K‐edge edge‐jump map of the OD image in Figure E–G) Bulk C K‐edge XANES of the presented edge‐jump map. H) Bulk XANES of the Cr L_2_ and L_3_ edge, extracted from Figure S2: PCA after removal of pixels with too much was impossible due to the low amount of Cr being atomically dispersed.

Figure 5 A shows a scanning electron microscopy (SEM) image of the pristine catalyst material, prior to treatment by a co‐catalyst and polymerization. The OD image at the O K‐edge (538 eV), in Figure 5 B, shows features resembling the pristine material and two clear phases are discerned, one corresponding to the SiO_2_ framework and one to the embedding epoxy resin. Clustering after principal component analysis (PCA) is reported in Figure 4 C and confirms the presence of three characteristic clusters, that is, regions with distinctly different spectral features (XANES). The corresponding normalized XANES at the O K‐edge of the clusters, shown in Figure 4 D, correspond to that of the epoxy resin and two clusters associated to the catalyst particle region of interest. In any case, the pre‐edge feature infers infiltration of the catalyst particle with the epoxy resin, since it should be absent in case of a pure SiO_2_ phase. With respect to the pristine Cr/SiO_2_ material, no carbonaceous material should be present within the catalyst, yet the carbon K‐edge edge‐jump map confirms its presence in the entire field of view, thus also the particle, with the corresponding carbon K‐edge bulk XANES (Figure 4 G) corresponding to the embedding epoxy resin.

In addition, Cr was found in the sample material, demonstrated by the bulk XANES of the Cr L_2,3_ edge in Figure 4 H. The ΔOD of ≈0.02 already corroborates the small amount of Cr atoms in the material cross‐section. The discrepancy between the OD values reported in Figure 4 H and the normalized absorption for the O K‐edge (Figure 4 D) comes from normalization of the XANES spectrum, with the Optical Density (before normalization of the O K‐edge XANES spectrum) shown in Figure S2, demonstrating that indeed the Cr L_2,3_ edge lies on the continuum of the O K‐edge. Naturally, increasing the cross‐section thickness would increase the ΔOD due to an increase in Cr atoms along the X‐ray path, this would unfortunately coincide with loss of spectroscopic information at the C K‐edge, due to absorption of nearly all the X‐rays in this case as well as the too large signal offsets due to high absorption. Furthermore, even with increased S/N values, the Cr sites are expected to be isolated and, at this cross‐section thickness, the isolated sites or multi‐atom ensembles are at least two orders of magnitude smaller than the spatial resolution of 100 nm.

**Figure 6:**
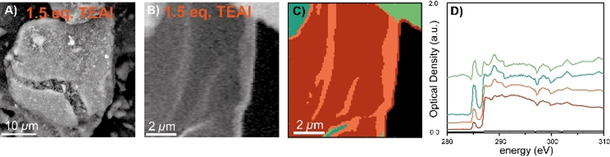
Results from the scanning transmission X‐ray microscopy (STXM) experiments before processing of the data. A) Scanning electron microscopy (SEM) image of the with 1.5 molecular equivalents TEAl pre‐polymerized catalyst particle. B) STXM optical density (OD) image at 280 eV of the cross‐section. C) Clustered image without applying edge‐jump analysis and normalization of the OD reporting image and D) corresponding raw XANES to the in Figure C presented.

Figure 6 A shows the SEM image of the pre‐polymerized catalyst particle, where the effect of polymerization on the particle morphology is visualized by the presence of extra surface features, attributed to PE, as well as by a significant crack formed due to disintegration of the SiO_2_ support particle due to increasing internal stress caused by the growing PE. Still, the particle morphology in Figure 6 A resembles that of the pristine material, thus confirming that fragmentation of the SiO_2_ support has not yet occurred extensively, meaning that the PE is still largely dispersed within the support phase. Figure 6 B reports an OD image at 285 eV of a cross section of this pre‐polymerized catalyst material. Where the top‐right and top‐left corners are brighter than the center of this image. Subsequent alignment of the stack and sample/background selection was performed in aXis2000, with PCA and clustering (without filtering of pixels based on edge‐jump analysis) being performed in TXM Wizard hereafter, the latter process producing the image in Figure 6 C and corresponding raw XANES in Figure 6 D. This shows five different regions, corresponding to in the OD image identifiable regions. Firstly, the top right corner is defined by absorption saturation, due to measuring very close (or partly on) the Cu TEM Grid. Secondly, the top‐left corner is less affected by absorption saturation, confirmed by much larger contrast and a peak intensity of ≈1 OD. The two residual clusters make up the center part of the OD image. The pre‐edge feature is attributed to the π*_C=C_ transitions of the epoxy resin whereas the features at 287.4 and 287.8 eV are attributed to PE σ*_C−H_ transitions. This means that the top‐left corner likely contains a larger contribution of the epoxy whereas the center region contains less of this and are predominantly occupied by PE.[^77^, ^80^]

**Figure 7:**
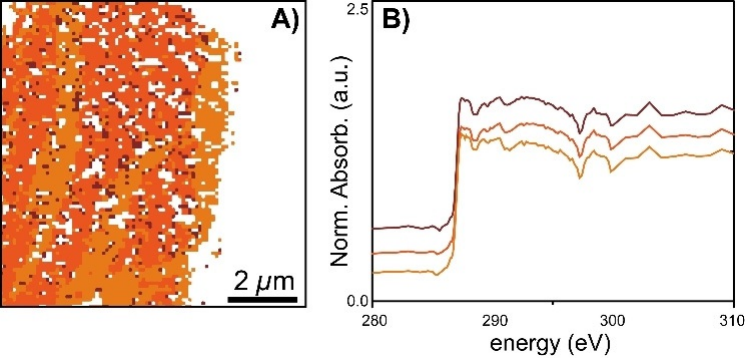
A) Clustered image after principal component analysis (PCA) of the with 1.5 molecular equivalents TEAl pre‐polymerized catalyst particle. The SEM image and optical density (OD) image are given in Figures 5 A and B respectively. B) C K‐edge X‐ray absorption near‐edge spectra (XANES) of clusters in the microtomed cross‐section of the catalyst material. Modified from as‐measured spectra of the indicated locations, by subtraction of the epoxy signal, according to the described procedure.

Take note, that for some of the experiments, specifically in Figures 6 D, 7 B, 9 D and 9 H, there appears to be two dips in the C K‐edge XANES at 297 and 300 eV: caused by the K 2p→3d spin‐orbit split transition. The materials produced with 1.5 and 5.0 mol. eq. of TEB and 1.5 mol. eq. of TEAl show these dips, whereas the sample with 5.0 mol. eq. TEAl does not. The latter was measured 9 months after the others, explaining why only one of the four signals does not contain the negative signals. However, the spectra are significantly similar to each other (and the reference materials) that it does not hamper analysis of the relevant signals at 287.4 and 287.8 eV.

**Figure 8:**
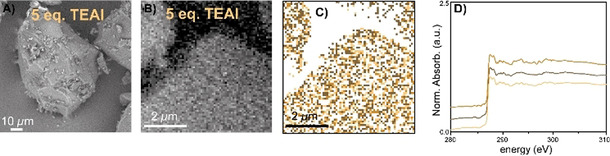
A) Scanning Electron Microscopy (SEM) image of the with 5.0 molecular equivalents tri‐ethyl aluminum (TEAl) pre‐polymerized catalyst material. B) Optical Density (OD) image at 280 eV of the microtomed cross‐section. C) Clustered image after Principal Component Analysis (PCA) at the C K‐edge of the microtomed cross‐section. D) C K‐edge X‐ray absorption near‐edge spectra (XANES) of the clusters in the microtomed cross‐section of the catalyst material. Modified from as‐measured spectra of the indicated locations, by subtraction of the epoxy signal, according to the described procedure.

**Figure 9:**
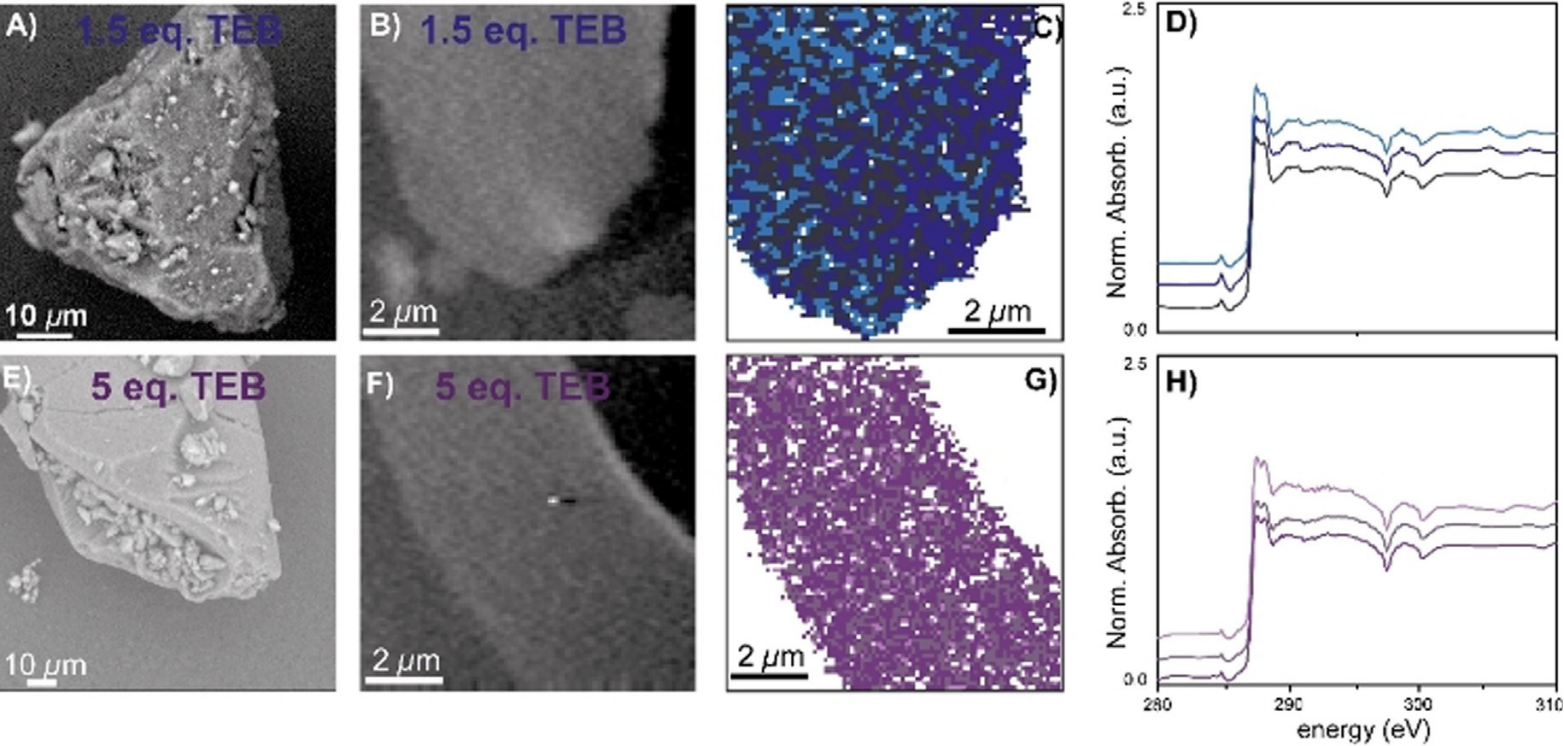
A) + E) Scanning electron microscopy (SEM) image of the with 1.5 and 5.0 molecular equivalents of tri‐ethyl borane (TEB) pre‐polymerized catalyst particle, respectively. B) + F) The optical density (OD) images at 280 eV of the microtomed cross‐section. C) Clustered image after principal component analysis (PCA) at the C K‐edge of the microtomed cross‐section. D) + H) C K‐edge X‐ray absorption near‐edge spectra (XANES) of the microtomed cross‐section of the catalyst material. Modified from as‐measured spectra of the indicated locations, by subtraction of the epoxy signal, according to the described procedure.

To solve the presence of mixed epoxy/PE phases, a method was developed that generated clustered images and C K‐edge XANES without epoxy contributions (Figure S4, Supporting Information provides individual steps): first, normal stack alignment and sample/background selection was performed using the aXis2000 software.^[92]^ Subsequently, PCA and clustering was performed using the TXM Wizard software.^[93]^ These two steps provided clustered images (with pixels pooled according to spectral similarity) and C K‐edge XANES with the epoxy contributor still present. The results are shown in Figure S5 for the materials produced with TEB and in Figure S6 for the materials produced with TEAl. The C K‐edge XANES of these materials show a pre‐edge feature at 285 eV, which indicates the presence of epoxy (see pure epoxy reference spectra in Figure S3). This feature is absent in the pure PE phase (see Figure 4) so it can be used as a quantitative marker for the presence of epoxy in each pixel by inspecting the magnitude of this feature in each normalized single‐pixel XANES. This allowed to effectively remove the contribution of pure epoxy to each spectrum: after normalization of every single pixel XANES the normalized reference of epoxy was weighted by the magnitude of the feature at 285 eV and subtracted [Eq. 1]:

(1) $$X_{i,corr}=X_{i}-w_{i}R_{epoxy}$$

where index *i* indicates the pixel index, *X*_*i*,corr_ the corrected XANES, *X_i_* the uncorrected XANES, *R* the epoxy reference, and w_*i*_ the weight for pixel I based on the magnitude of the 285 eV feature recorded for that pixel and scaled between 0 and 1; *w_i_*=1 indicates a pure epoxy spectrum based on the magnitude of the 285 eV feature in the epoxy reference and *w_i_*=0 the absence of any contribution from epoxy. The effectiveness of this method is nicely confirmed by the fact that all XANES of pixels containing pure epoxy have been reduced to their baseline and subsequently removed by the edge jump filter. After this removal of contributions from epoxy, PCA and clustering were again performed to group pixels according to their spectral similarity. The results are shown in Figure 7 and Figure 8 for the materials pre‐polymerized with TEAl and in Figure 9 for the materials pre‐polymerized with TEB.

Figure 8 A demonstrates the early‐stage polymer materials with 5.0 mol. eq. of TEAl, respectively. Again, this material is characterized by its largely intact original particle morphology in combination with starting cracks and surface features, with the original particle morphology being retained to a large extent: indicating that this particle is still in the early stages of disintegration.

The OD images are shown in Figures 6 B and 8 B. In the case of the OD image shown in Figure 6 B, which was discussed before, one can clearly differentiate between the different regions in the catalyst material. The OD image of the cross‐section with 5.0 mol. eq. of TEAl, shown in Figure 8 B, does not clearly show an epoxy(‐rich) region or PE(‐rich) region, instead the pre‐polymerized catalyst particle cross‐section occupies the entire field of view. However, Figure S6G clearly shows epoxy throughout the catalyst material. The image sequence in Figure 8 was collected with a smaller field of view of 7×7 μm while maintaining the same pixel resolution of 100 nm.

Clustering after PCA with the epoxy removed by linear subtraction further confirmed the successful removal of the epoxy contributions, as shown in Figures 7 A and 8C, which is further corroborated by the C K‐edge XANES in Figures 7 B and 8D, now free of epoxy contributions. The remaining clusters in Figure 7 A now perfectly overlap with the early‐stage catalyst material shown in the OD image in Figure 6 B. These clusters are internally characterized by similar 287.4 and 287.8 eV σ*_C−H_ absorption features and 290–292 eV σ*_C−C_ absorption feature at the C K‐edge XANES, which indicate only minor differences between the clusters. Interestingly, there appears to be at least some spatial correlation within this sample coinciding with the OD image, which are related to slight variations in sample thickness: the brighter parts corresponding to more absorption (more material) and the less bright residual volume of the catalyst cross‐section is slightly less absorbing.

A comparison of the C K‐edge XANES of the materials produced with 1.5 mol. eq. TEAl (Figure 7 B) and 5.0 mol. eq. of TEAl (Figure 8 D) demonstrates that the line shapes of the 287.4 and 287.8 eV σ*_C−H_ absorption features of these materials are relatively similar, with the individual transitions being slightly more obscured for the material produced with 1.5 mol. eq. TEAl (Figure 7 B). Furthermore, Figures 7 B and 8D in both cases demonstrate similar σ*_C−C_ absorption features in the 290–292 eV range. These observations show that these materials are similar in terms of density, albeit this likely being slightly lower for the material produced with 1.5 mol. eq. In accordance with the bulk‐investigations, the larger density for the material with 5.0 mol. eq. TEAl is possibly explained by an expectedly relatively larger number of smaller PE molecules co‐crystallizing between bigger PE chains.[^78^, ^81^]

Figure 9 demonstrates the results of the STXM measurements with TEB as a co‐catalyst, in which Figures 9 A and 9 E show the SEM images of the pre‐polymerized catalyst particles. Here, the early‐stage of ethylene polymerization is confirmed by the presence of additional surface features and the absence of extensive catalyst fragmentation. The SEM images clearly show that the original particle morphology is largely retained.

Figures 9 B and F show the OD images at the C K‐edge. Clearly the catalyst material can be discerned from the Struers Epofix epoxy resin in both cases: the darker areas corresponding to the epoxy resin and the lighter material to the pre‐polymerized catalyst material. Clustering after PCA, as shown in Figures 9 C and G, with three clusters (corresponding XANES in Figure 9 D and H) show that the regions associated to epoxy are correctly subtracted, now revealing pure PE spectra.

First, the XANES of the early‐stage PE material produced with 1.5 mol. eq. of TEB, shown in Figure 9 D, is characterized by two distinct σ*_C−H_ X‐ray absorption features at 287.4 and 287.8 eV. In case 5.0 mol. eq. TEB was used, the produced XANES in Figure 9 H, demonstrate a similar line shape: with clear and individual transitions at 287.4 and 287.8 eV.

On first sight, it appears that internally the growth of PE is homogeneous in each sample, testified by the similar XANES for the different clusters and further corroborated by Figures 9 C–D and G–H that exhibit little spatial hierarchy. Having said this, Figure 9 C appears to demonstrate some spatial hierarchy inferred by a larger concentration of „dark‐blue“ clusters at the right side and a larger concentration of „light‐blue“ clusters at the left side. However, as mentioned before, the C K‐edge strongly resemble each other and likely infer homogeneous PE formation throughout the catalyst cross‐section, thus if these PEs differ, it is only very minimally.

**Figure 10:**
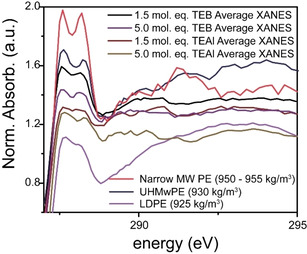
Magnification of the 287*–*295 eV region in the C K‐edge X‐ray absorption near‐edge spectra (XANES) of the average spectra of the early‐stage polyethylene (PE) materials as well as the spectra of three relevant reference materials. The C K‐edge XANES are offset for clarity.

A quick comparison of the materials produced with TEB to those produced with TEAl (magnification shown in Figure 10), shows that splitting of the characteristic 287.4 and 287.8 eV σ*_C−H_ X‐ray absorption features is clearly present for the materials produced with 1.5 mol. eq. and 5.0 mol. eq. TEB and for the material produced with 5.0 mol. eq. TEAl. The splitting can still be distinguished for the material produced with 1.5 mol. eq. TEAl, however it can be argued that it is more obscured here. These results infer that the PE materials produced with 1.5 and 5.0 mol. eq. of TEB and the material produced with 5.0 mol. eq. of TEAl are very similar in terms of density, whereas the density of the material produced with 1.5 mol. eq. of TEAl is likely a bit lower.[^77^, ^82^]

### Relating bulk characterization data to the X‐ray imaging technique

Figure 10 also allows us to easily compare the C K‐edge XANES of three reference materials, the two outliers (LDPE and Narrow MW PE) and the UHMwPE, with the averaged C K‐edge XANES of the early‐stage PE materials.

As mentioned, the early‐stage PE are very similar in terms of density on basis of their 287.4 and 287.8 eV σ*_C−H_ X‐ray absorption features, which all show at least some splitting, with the splitting for the material produced with 1.5 mol. eq. TEAl being the most obscured of the four averages. One of the underlying reasons for this might be the increased SCB lowering the density. Figure 10 also shows that the early‐stage PE produced with 5.0 mol. eq. of TEAl demonstrates a slightly more pronounced splitting of the 287.4 and 287.8 eV signals, likely due to the broader MWD: a relatively larger amount of small PE chains exist in this product, which can co‐crystallize between the bigger chains, hereby increasing the PE density, also previously discussed.^[81]^

What is evident form Figure 10 is that the C K‐edge XANES of none of the early‐stage materials approaches the C K‐edge XANES of the 950–955 kg m^−3^ reference, instead, the materials are more comprable to the 930 kg m^−3^ reference material. This indicates that the density of the early‐stage materials is lower than what is to be expected for HDPE materials.

This finding coincides with findings by McKenna et al., reporting that nascent early‐stage PE materials demonstrated lower melting temperatures (*T*_m_) than was expected. This was attributed to the PE crystallite size dimensions being in line with the pore size dimensions of the original catalyst material, hereby confining polymer growth.[^83^, ^84^, ^85^] Similarly, we used DSC (Table S4, Figures S11 and S12) to assess the *T*_m_ of the early‐stage materials and in general found that these are lower than those of bulk PE materials (≈135 °C). More specifically, the early‐stage PE materials produced with 1.5 mol. eq. of TEAl demonstrate melting temperatures at 119.05 and 132.7 °C and for 5.0 mol. eq. TEAl melting temperatures of 120.9, 130.2 and 136.8 °C are measured. For TEB, the melting temperatures were 127.02 and 136.07 °C for the PE produced with 1.5 mol. eq. TEB and 121.2 °C for the PE produced with 5.0 mol. eq. of TEB. The multiple *T*_m_ for the materials produced with TEAl and 1.5 mol. eq. TEB are likely caused by the presence of different defective sequences, which constitute crystals of different thickness and is a testament to the presence of relatively larger amounts of SCB.[^4^, ^71^, ^83^, ^84^, ^85^, ^86^, ^87^]

The DSC results support the STXM results in a sense that indeed we see that the early‐stage PE materials have lower densities than bulk HDPE materials, emphasizing the effect of the support matrix.[^83^, ^85^] This is often explained due to PE materials with higher degrees of SCB being less crystalline while still dispersed in the SiO_2_ framework and are proposed to be able to diffuse out of the SiO_2_ pores, whereas the more crystalline and rigid PE materials are less able to do so.[^84^, ^86^, ^87^, ^88^]

## Conclusions

In this work we found that the properties of the polyethylene (PE) produced by a single Cr/SiO_2_ Phillips catalyst formulation can be carefully tailored by selection of the proper type and amount of tri‐ethyl borane (TEB) or tri‐ethyl aluminum (TEAl). More specifically, PE properties in terms of molecular weight distribution (MWD), short‐chain branching (SCB) and long‐chain branching (LCB) were found to be sensitive to this and could be tuned. Both co‐catalysts broadened the MWD, however TEB did so to a greater extent than TEAl. TEAl, in turn, was more beneficial for SCB than TEB and exhibited a larger degree of SCB. For both co‐catalysts, only minor deviations from the theoretical linear reference in terms of viscosity are observed, excluding the PE materials produced with 4.86 mol. eq. and 11.72 mol. eq. TEB (respectively 1.50 and 3.00 ppm), here at least some deviations were observed.

Scanning transmission X‐ray microscopy (STXM) measurements on the effect of TEB and TEAl on the nanometer‐scale PE density and related crystallinity revealed that within one catalyst material the growth of PE is homogeneous, testified by only minor differences within the C K‐edge XANES. Furthermore, we found that the densities of the early‐stage materials were very similar. However, it could be argued that the densities for the early‐stage materials produced with TEAl were slightly lower: the material produced with 1.5 mol. eq. of TEAl being the smallest. A comparison with the reference materials demonstrated overall smaller densities than what was to be expected for HDPE bulk materials, this being further corroborated by the *T*_m_ from the DSC measurements.

In summary, we have shown that STXM is a powerful tool in characterizing these early‐stage PE materials. We have shown that both TEB and TEAl distinctly affect the early‐stage PE and we have shown that the early‐stage PE materials are, type‐wise, different than their bulk variants, indicating the significance of the support in confining the early‐stage PE by affecting the density and related crystallinity.

## Experimental Section

**Batch‐reactor catalyst testing**: Slurry phase polymerization reactions with a Cr/SiO_2_ Phillips‐type catalyst were performed in a 5 L semi‐batch reactor at SABIC Geleen, during which induction time, catalyst yield and total polymerization time were monitored. The batch reactor was loaded with 1071 g isobutane (SABIC, 96 %, 4 % n‐butane) as diluent and heated to 99 °C. 830 mg H_2_ (SABIC, 99.9 %) was added and subsequently the reactor was pressurized to 34 bar with C_2_H_4_ (SABIC, 99.9 %). Hence, the diluent was loaded with 12 mol % C_2_H_4_ and 1 mol % H_2_. Upon reaching the reaction conditions the co‐catalyst was injected with 120 g of isobutane and subsequently the Cr/SiO_2_ catalyst was injected with 180 g of isobutane. Ethylene was fed to the reactor to maintain constant pressure. A catalyst yield of approximately 2700 g of polyethylene per g of catalyst was used as an end‐point of the reaction. The co‐catalysts under study are: tri‐ethylborane (TEB, SABIC, 99 %) and tri‐ethylaluminum (TEAl, SABIC, 99.0 %). The Cr/SiO_2_ is a silica Cr‐catalyst with a ≈1.0 wt % Cr loading, a surface area of 625 m^2^ g^−1^, a pore volume of 2.41 mL g^−1^ nad a D50 particle size distribution of 52.8 μm. The catalyst was calcined at 650 °C via a SABIC‐propriety technique to yield the used CrO_x_/SiO_2_ catalyst.

**Nuclear magnetic resonance**: The samples were dissolved at 130 °C in C_2_D_2_Cl_4_‐containing di‐*tert*‐butyl‐*p*‐cresol (DBPC, Sigma–Aldrich, >99 %) as stabilizing agent. The ^13^C NMR spectra were recorded on a Bruker Avance500 spectrometer with a cryogenically cooled probe operated at 125 °C.

**Size exclusion chromatography–differential viscometry–infrared measurements**: SEC‐DV‐IR was carried out on a PolymerChar GPC‐IR system running at 160 °C equipped with a Polymer Char IR5 infrared detector and a PolymerChar viscometer. The column set consisted of three Polymer Laboratories 13 um PLgel Olexis 300×7.5 mm columns. PE molar mass calibration was performed with linear PE standards in the range of 0.5–2800 kg mol^−1^ (Mw/Mn=4 to 15).

**Scanning electron microscopy**: SEM micrographs of the early‐stage polyethylene materials were recorded on a PhenomPro X microscope (FEI Company, USA), equipped with a CsB detector for backscattered electrons (BSE), operated at 10 kV. The samples were deposited onto Al stabs with carbon tape (electron Microscopy Sciences, Hartfield, PA, USA) that were not coated prior to measurements.

**Scanning transmission X‐ray microscopy**: The sample preparation for STXM experiments was performed by embedding product powders after polymerization in a quartz cell, pre‐treated with either 1.50 and 5.00 mol. eq. of TEB or TEAl in a Struers Epofix epoxy resin. The embedded material was ultra‐microtomed with a Diatome Ultra 35° diamond knife on a Reichert‐Jung Ultracut E into 100 nm slices and floated on a bath of water, after which they were placed on transmission electron microscopy (TEM) grids and used as such for analysis. The STXM measurements were performed at the advanced light source (ALS) beamline 11.0.2, USA, and at the PolLux STXM of the swiss light source (SLS),[^89^, ^90^, ^91^] Paul Scherrer Institute, Switzerland. For the measurements across the C (275–320 eV), O (525–580 eV) and Cr (560–610 eV) edge a 45 nm Au zoneplate was used at the ALS and a 35 nm Au zoneplate was used at PolLux. The X‐rays at the ALS were circularly polarized to avoid contrast from dichroism and the X‐rays at the SLS are 80 % horizontal linear polarization. The probed area of the particles varied between 50 and 100 μm^2^ with a pixel size of 100 nm. The energy scan step size varied from 0.1 eV around the absorption edge to 1 eV in the pre‐edge and post‐edge regions.

**Scanning transmission X‐ray microscopy data analysis**: Data analysis was performed with the aXis2000^[92]^ and TXM‐XANES‐Wizard^[93]^ software. The alignment of the different image sequences was done in aXis2000, after which all spectra were converted to optical density (OD). Principal component analysis (PCA) analysis and clustering was performed with the TXM‐XANES‐Wizard software. First, pixels with X‐ray absorption edge jumps below a certain threshold were filtered out as they did not show sufficient S/N ration for further processing (especially normalization). After XANES normalization (here again single pixel spectra that showed insufficient quality for normalization were filtered) PCA and k‐means clustering was performed keeping three Principal Components (PC) for clustering in PC space. The number of clusters was selected manually based upon inspection of the results from PCA (eigenspectra, eigenimages, and score plot). Subsequently, de‐mixing of the XANES by removal of the contribution of the epoxy XANES was performed by subtracting an epoxy reference XANES from each single pixel XANES after weighting it by the peak area of the 285 eV X‐ray absorption feature, a feature that is exclusively present in the epoxy XANES. The corrected data set was again processed as before (normalization, filtering, PCA, k‐means clustering in 3‐dimensional PC space with manually selected number of clusters (typically 3)).

**Nitrogen physisorption**: N_2_ adsorption isotherms were measured at 77 K on a Micromeritics TriStar 3000 instrument. Prior to all measurements, samples were dried at 423 K under dynamic vacuum. Specific surface areas (SSAs) were calculated using the multipoint BET method (0.05<p/p0 <0.25). Pore volumes (Vp) were calculated by the t‐plot method; pore size distributions (PSDs) were obtained by DFT using N_2_ and spherical pores in the package MicroActive 4.06 (Micromeritics).

**Differential scanning calorimetry**: DSC was performed on a TA Instruments DSC Q20 with 1–2 mg of the nascent material. Each sample was heated from −40 °C to 200 °C at a rate of 10 °C min^−1^ after which it was briefly held isothermally at 200 °C. Subsequently the cooling cycle was initiated to −40 °C at a rate of 10 °C min^−1^ followed by an additional heating cycle to 200 °C at a rate of 10 °C min^−1^. The crystallinities of the polyethylene materials were determined assuming *ΔH*_m_^0^=293 J g^−1^ for 100 % crystalline polyethylene. The calculated crystallinities were not corrected for the residual amount of catalyst.

## Conflict of interest

The authors declare no conflict of interest.

## Supporting information

As a service to our authors and readers, this journal provides supporting information supplied by the authors. Such materials are peer reviewed and may be re‐organized for online delivery, but are not copy‐edited or typeset. Technical support issues arising from supporting information (other than missing files) should be addressed to the authors.

Supplementary
