# Supplementary material for: Influence of Metal‐Alkyls on Early‐Stage Ethylene Polymerization over a Cr/SiO2 Phillips Catalyst: A Bulk Characterization and X‐ray Chemical Imaging Study
Source: Chemistry. 2020 Dec 9;27(5):1688–99. doi: 10.1002/chem.202002632 (PMC7898848; doi:10.1002/chem.202002632)
Supplement: Supplementary file 1 — Supplementary [file CHEM-27-1688-s001.pdf]

# Chemistry–A European Journal

Supporting Information


## Influence of Metal-Alkyls on Early-Stage Ethylene Polymerization over a $Cr/SiO_2$ Phillips Catalyst: A Bulk Characterization and X-ray Chemical Imaging Study


Maarten K. Jongkind,[a] Florian Meirer,[a] Koen W. Bossers,[a] Iris C. ten Have,[a] Hendrik Ohldag,[c, d, e] Benjamin Watts,[f] Theo van Kessel,[b] Nic. Friederichs,[b] and Bert M. Weckhuysen*[a]

**Table S1.** Used energy profile for the scanning transmission x-ray microscopy measurements along the C K-edge.

| | |
|---|---|
| 284 – 286 | 0.2 |
| 286 – 290 | 0.1 |
| 290 – 300 | 0.2 |
| 300 – 320 | 1.0 |

**Table S2.** Used energy profile for the scanning transmission x-ray microscopy measurements along the Cr $L_{2,3}$-edge.

| Energy (eV) | Stepsize |
|---|---|
| 565 – 575 | 1.0 |
| 575 – 591 | 0.5 |
| 591 – 602 | 1.0 |
| 602 – 620 | 2.5 |

**Table S3.** Used energy profile for the scanning transmission x-ray microscopy measurements along the O K-edge.

| Energy (eV) | Stepsize |
|---|---|
| 520 – 530 | 1.0 |
| 530 – 551 | 0.5 |
| 551 – 575 | 1.0 |

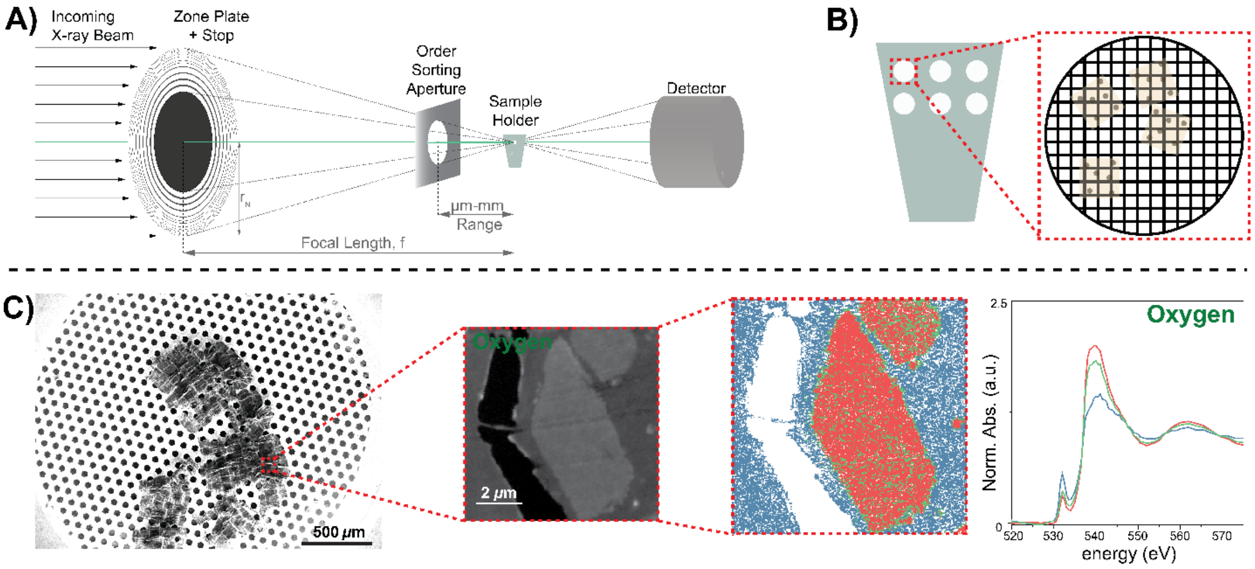


**Figure S1. A)** Figure **S1** schematically illustrates the principles in scanning transmission x-ray microscopy (STXM), in which incident X-ray beams were focused by a 37nm gold zone plate, passing through the Order Sorting aperture which filtered zero order, unfocused light, and subsequently passing through the sample and reaching the detector. **B)** The materials under investigation consisted of 100 nm thick coupes of the pre-polymerized catalyst particles in Struers Epofix Epoxy resin deposited on uncoated Cu TEM grids, which were loaded on the STXM holder and used as such. **C)** Scanning Electron Microscopy (SEM) image of the microtomed coupes on the TEM grid. Furthermore, optical density (OD) image at the oxygen K-edge, 580 eV, reveals two distinct materials: epoxy resin and $SiO_2$ which are identifiable in the clusters after Principal Component Analysis (PCA) in combination with the respective XANES of the two clusters.

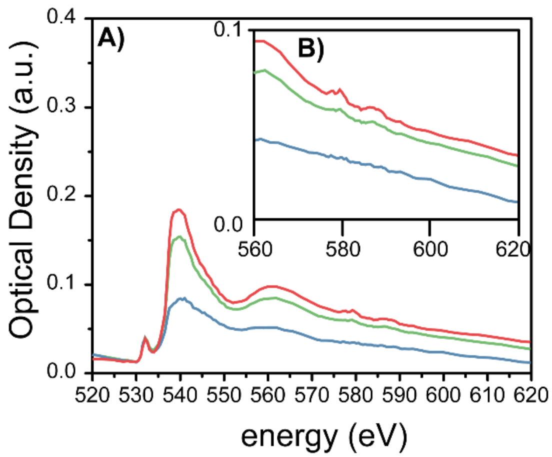


**Figure S2. A)** Non-normalized XANES of the clusters at the Oxygen K-edge. Demonstrating that the Cr $L_{2,3}$ edge lies on the continuum of the O K-edge. **B)** Magnification of the Cr $L_{2,3}$ edge in the 570 – 620 eV region. The blue XANES corresponds to the background region: that is the region without catalyst material. The red and green lines correspond to catalyst material regions: the averaged spectrum in Figure **5H** is extracted from this.

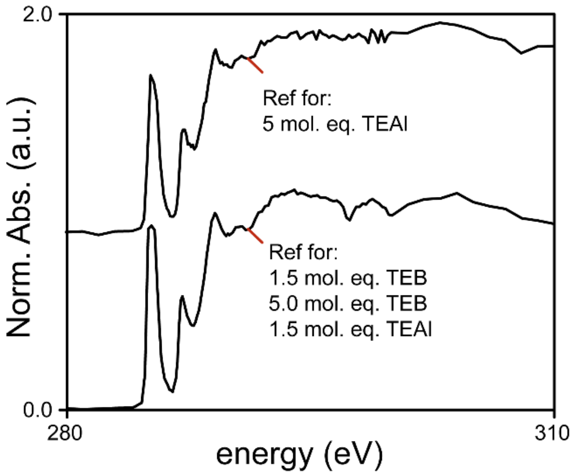


**Figure S3.** Normalized Epoxy reference XANES measured at ALS Beamline 11.0.2 and used, as such, for subtraction from the combination XANES by LCA. The spectra are offset for clarity.

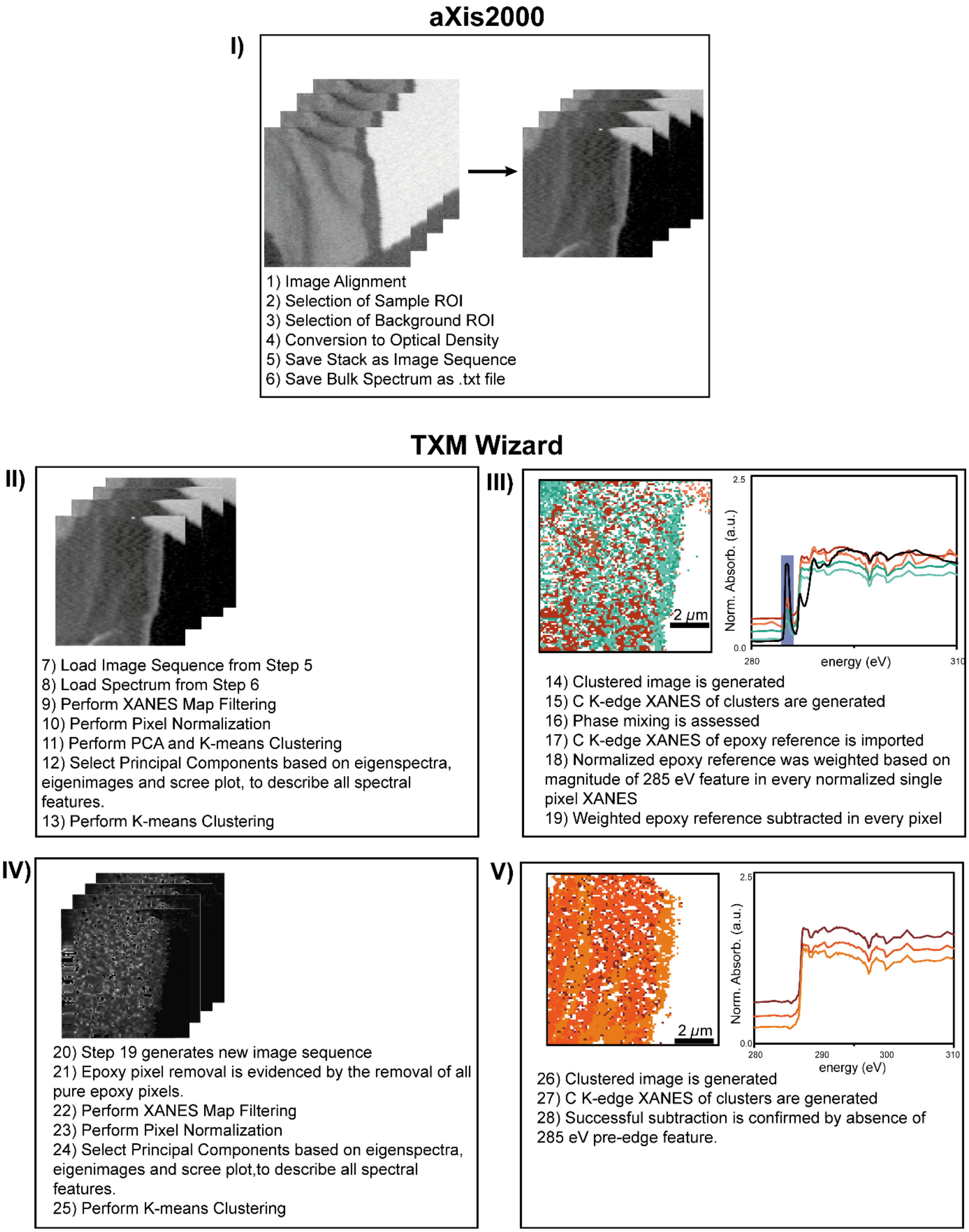


**Figure S4.** Illustration describing all individual steps of the data processing.

**I)** Data analysis started with the raw data obtained from the beamline. It was opened in the aXis2000[1] software package and it was converted into a .ncb file. Subsequently, the “Jacobsen Stack Analyze” function was used for aligning the images in the stack based on each previous image. The aligned stack was saved as a .ncb file. Hereafter, the “Stack Analyze” function in the aXis2000 software package was used to open the aligned stack, where the regions of interest (ROI) of the void space (background, I0) and sample (rest, I) were selected and the stack was converted to Optical Density (OD). The stack was saved, along with the individual images at each energy point (.tif) and a Bulk XANES spectrum.

**II)** The previously generated image sequence (.tif) was loaded in TXM Wizard. Noise filtering is performed based on the edge jump and normalization quality of every single pixel XANES to remove pixels that show insufficient signal to noise region. A detailed explanation is given in Y. Liu, F. Meirer *et al*.[2] Hereafter, PCA and K-means clustering was performed to pool clusters based on spectral similarities. The number of Principal Components was selected based upon inspection of the scree plot, the eigenspectra and eigenimages. Hereafter, K-means clustering was performed using a number of clusters that was initially based on the number of PC kept and refined upon inspection of the obtained XANES of the generated clusters.

**III)** The clustered image was now generated, along with the C K-edge XANES of the individual clusters. This allowed to localize both pure epoxy components as well as mixed phases in the Field of View. Hereafter, a normalized reference XANES of the pure epoxy resin was loaded and compared to the XANES of the clusters. The pure epoxy resin contains a pre-edge feature at 285 eV that is absent for PE materials. Therefore, it can be used as a quantitative marker for the presence of epoxy in each pixel by inspecting the magnitude of this feature in each normalized single-pixel XANES. This reference for pure epoxy resin was weighted by the magnitude of the feature at 285 eV in the XANES of each pixel and subtracted according to the formula:

$$X_{i,corr} = X_i - w_i R_{epoxy}$$

Where index *i* indicates the pixel index, $X_{i,corr}$ the corrected XANES, $X_i$ the uncorrected XANES, R the epoxy reference, and $w_i$ the weight for pixel i based on the magnitude of the 285 eV feature recorded for that pixel and scaled between 0 and 1; $w_i$ =1 indicates a pure epoxy spectrum based on the magnitude of the feature in the epoxy reference and $w_i$=0 the absence of any contribution from epoxy. The effectiveness of this method is confirmed by the fact that all XANES of pixels containing epoxy have been reduced to their baseline and subsequently removed by the edge jump filter.

**IV)** This step generated a new image sequence: the pixels that contained pure epoxy were removed and the epoxy contribution in mixed phase pixels has now been removed. Hereafter, the same procedure for normalization and clustering as described above was applied.

**V)** The clustered image was generated along with the related C K-edge XANES. The C K-edge XANES showed the successful removal of the 285 eV feature as well as pure PE components.

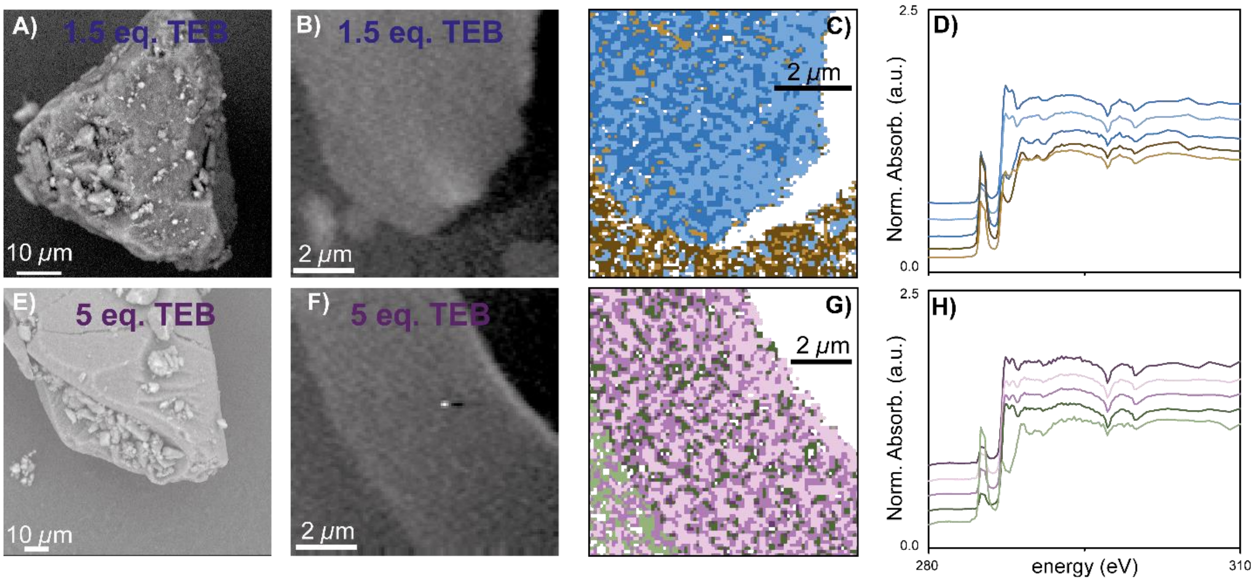


**Figure S5.** Overview of the Scanning Transmission X-ray Microscopy (STXM) measurement results on the with 1.5 and 5.0 mole equivalents of tri-ethyl borane (TEB) pre-polymerized Cr/$SiO_2$ catalyst particles before linear subtraction of the proper epoxy reference C K-edge XANES. **A and E)** Scanning Electron Microscopy (SEM) images of the pre-polymerized catalyst materials with respectively 1.50 and 5.0 mole equivalents of tri-ethyl borane (TEB). **B and F)** STXM optical density (OD) image at the C K-edge (280 eV) of the microtomed slice of the pre-polymerized material. **C and G)** Clustered image after Principal Component Analysis (PCA) on the stacks obtained at the C K-edge, showing distinct phases: carbon from the Struers Epofix Epoxy resin and carbon from the polyethylene. **D and H)** Obtained C K-edge XANES of the clustered images.

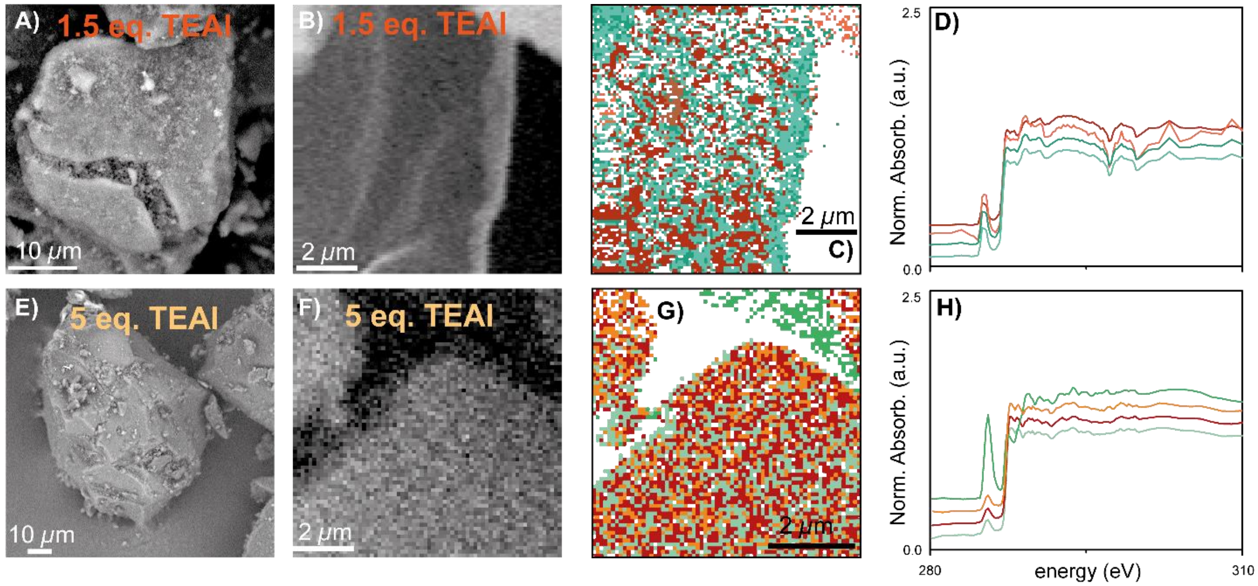


**Figure S6.** Overview of the Scanning Transmission X-ray Microscopy (STXM) measurement results on the with 1.5 and 5.0 mole equivalents of tri-ethyl aluminum (TEAl) pre-polymerized Cr/$SiO_2$ catalyst particles before linear subtraction of the proper epoxy reference C K-edge XANES. **A and E)** Scanning Electron Microscopy (SEM) images of the pre-polymerized catalyst materials with respectively 1.50 and 5.0 mole equivalents of tri-ethyl borane (TEB). **B and F)** STXM optical density (OD) image at the C K-edge (280 eV) of the microtomed slice of the pre-polymerized material. **C and G)** Clustered image after Principal Component Analysis (PCA) on the stacks obtained at the C K-edge, showing distinct phases: carbon from the Struers Epofix Epoxy resin and carbon from the polyethylene. **D and H)** Obtained C K-edge XANES of the clustered images.

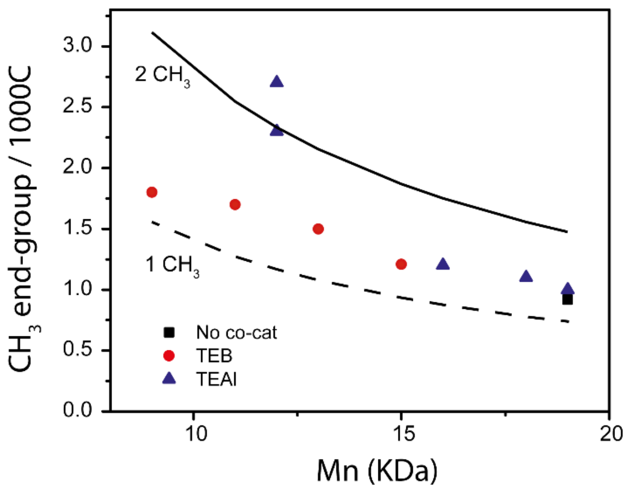


**Figure S7.** $CH_3$ end-groups plotted versus the $M_n$. Additionally, the theoretical lines for 2 $CH_3$ end-group per PE chain and 1 $CH_3$ end-group per PE chain are plotted for assessing how much $CH_3$ groups per PE chains are present.

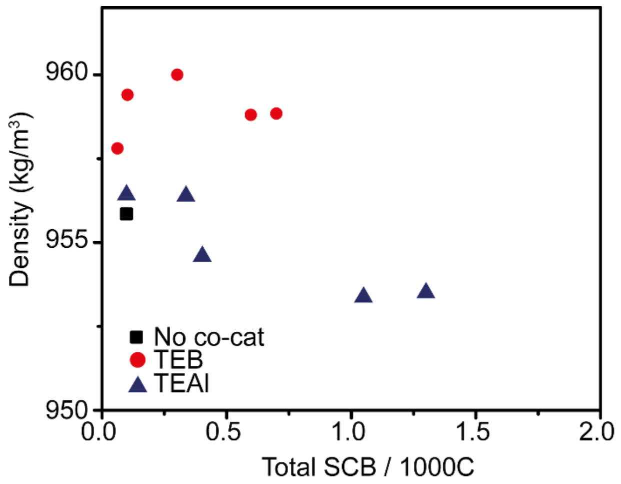


**Figure S8.** The densities of the produced PE materials plotted versus the cumulative number of Short Chain Branches (SCB).

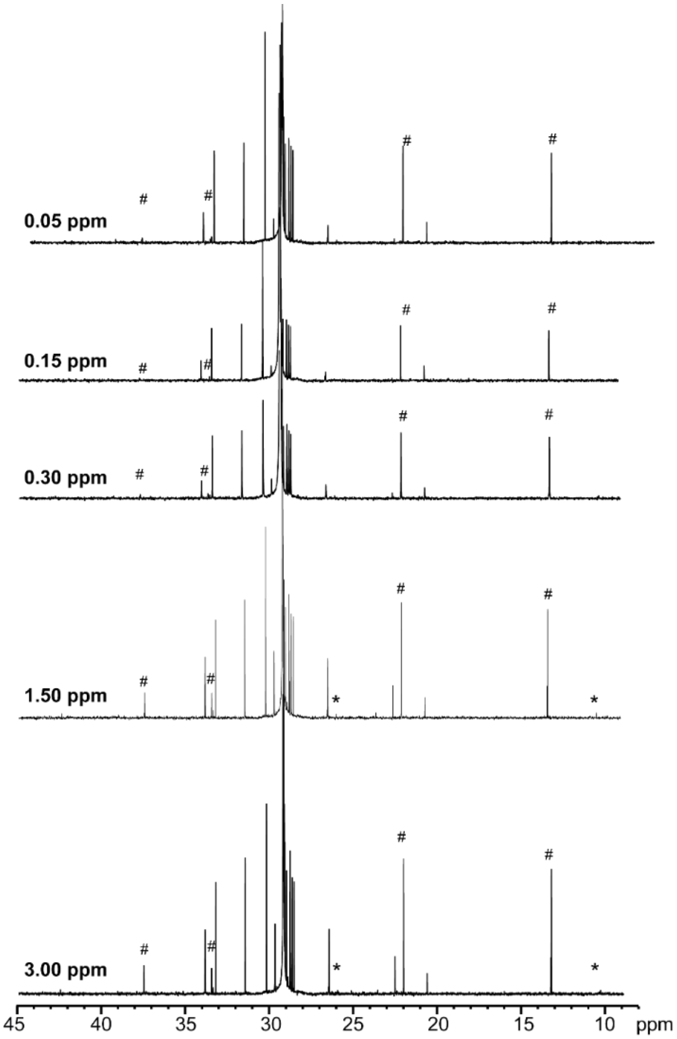


**Figure S9.** $^{13}C$-Nuclear Magnetic Resonance (NMR) spectra of the polyethylene powders produced with 0.05, 0.15, 0.30, 1.50 and 3.00 ppm tri-ethyl borane (TEB) in the 5L semi-batch ethylene polymerization reactions. Increasing amounts of TEB result in larger amounts of butyl (#) and ethyl (*) short chain branches.

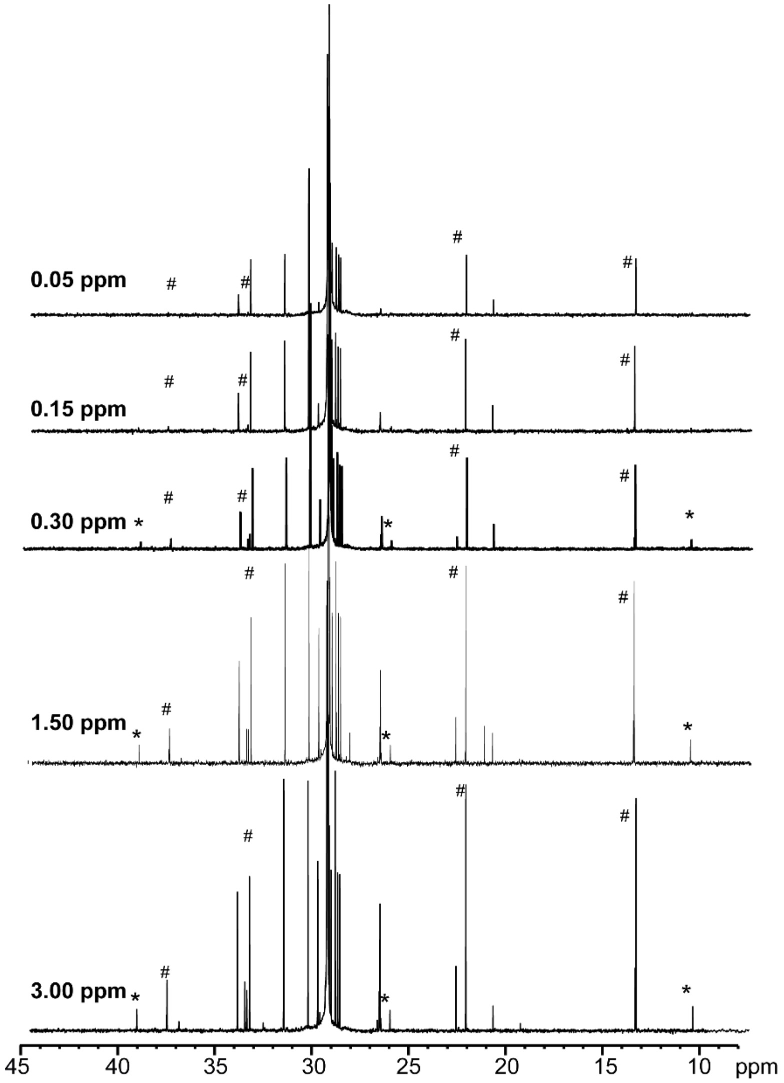


**Figure S10.** $^{13}$C-Nuclear Magnetic Resonance (NMR) spectra of the polyethylene powders produced with 0.05, 0.15, 0.30, 1.50 and 3.00 ppm tri-ethyl aluminum (TEAl) in the 5L semi-batch ethylene polymerization reactions. Increasing amounts of TEB result in larger amounts of butyl (#) and ethyl (*) short chain branches.

Differential Scanning Calorimetry (DSC) was performed on a TA Instruments DSC Q20 with 1-2 mg of the nascent material. Each ample was heated from -40 °C to 200 °C at a rate of 10 °C min$^{-1}$ after which it was briefly held isothermally at 200 °C. Subsequently the cooling cycle was initiated to -40 °C at a rate of 10 °C min$^{-1}$ followed by an additional heating cycle to 200 °C at a rate of 10 °C min$^{-1}$. The crystallinities of the materials, as shown in Table **S4**, were determined assuming $\Delta H_m^0$ = 293 J/g for 100% crystalline polyethylene, with the fraction of the measured $\Delta H_m$ representing the crystallinity of the nascent early stage materials. The residual catalyst masses were not taken into account, resulting in a significant underestimation of the crystallinity. Due to possible variations in PE yields, we refrained from a discussion on the crystallinity in the main text. Yield variations in 1-1.5 g/g already result in 0-50% differences in the determined crystallinity.

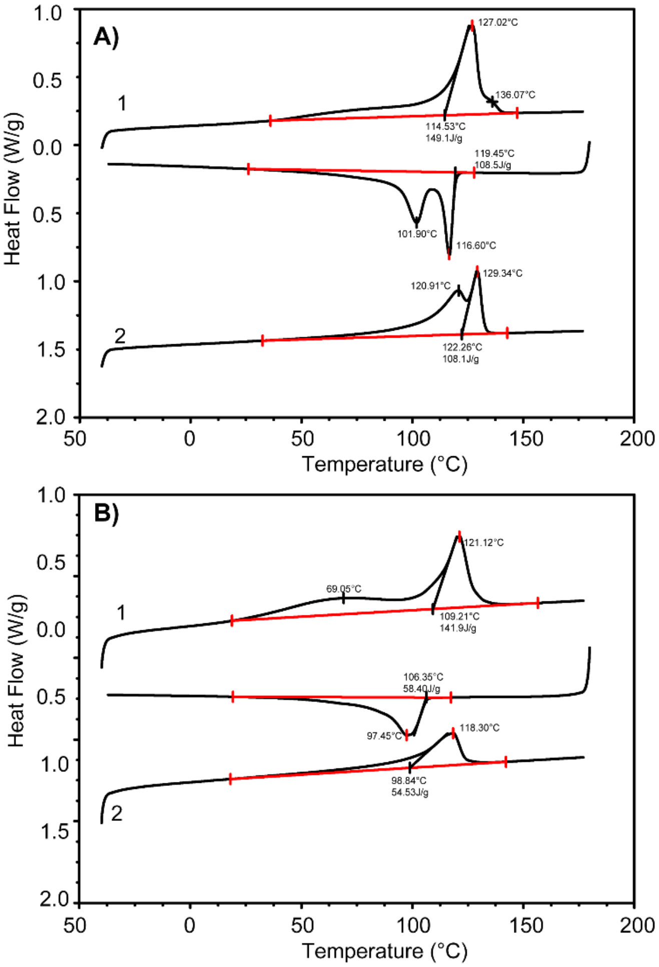


**Figure S11.** Differential Scanning Calorimetry (DSC) profiles for the Early-Stage polyethylene Materials produced with **A)** 1.5 mole equivalents and **B)** 5.0 mole equivalents of tri-ethyl borane (TEB). The first heating ramp was from -40 °C to 180 °C with 10 °C/min, after which the temperature was kept constant at 180 °C to erase the thermal history. Subsequently the sample was cooled from 180 °C to -40 °C with a ramp of 10 °C/min and the second heating cycle was started from -40 °C to 180 °C with a 10 °C/min ramp.

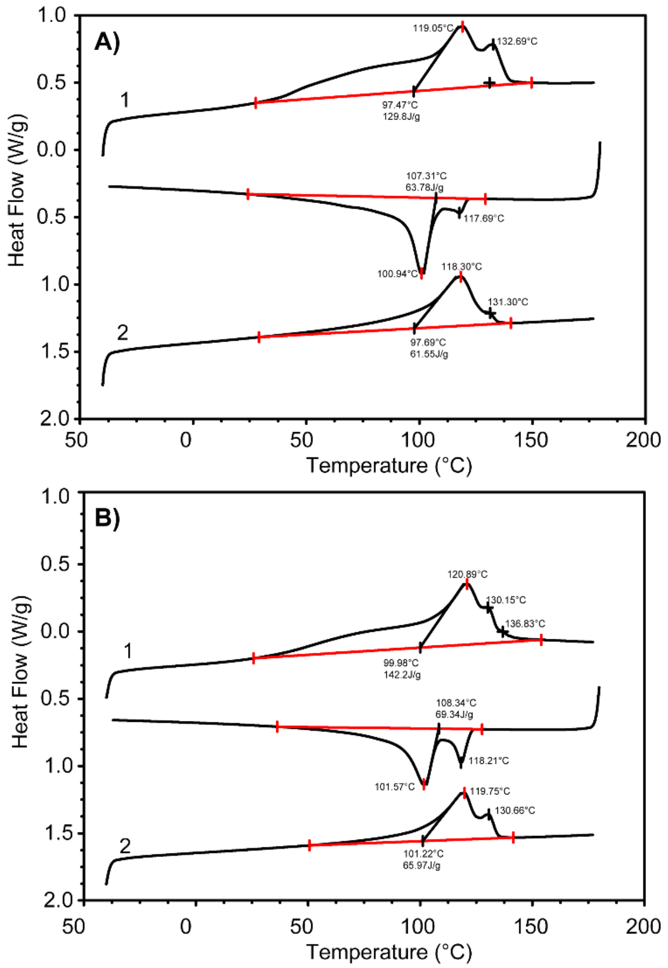


**Figure S12.** Differential Scanning Calorimetry (DSC) profiles for the Early-Stage polyethylene Materials produced with **A)** 1.5 mole equivalents and **B)** 5.0 mole equivalents of tri-ethyl aluminum (TEAl). The first heating ramp was from -40 °C to 180 °C with 10 °C/min, after which the temperature was kept constant at 180 °C to erase the thermal history. Subsequently the sample was cooled from 180 °C to -40 °C with a ramp of 10 °C/min and the second heating cycle was started from -40 °C to 180 °C with a 10 °C/min ramp.

**Table S4.** Crystallinities of the early-stage PE materials obtained by Differential Scanning Calorimetry (DSC).

| Sample | $X_c$ (%) | $T_{m1}$ (° C) |
|---|---|---|
| **TEB** | | |
| 1.50 eq. | 50.9 | 127.02; 136.07 |
| 5.00 eq. | 48.4 | 121.2 |
| **TEAL** | | |
| 1.50 eq. | 44.3 | 119.05; 132.7 |
| 5.00 eq. | 48.5 | 120.89; 130.15; 136.83 |

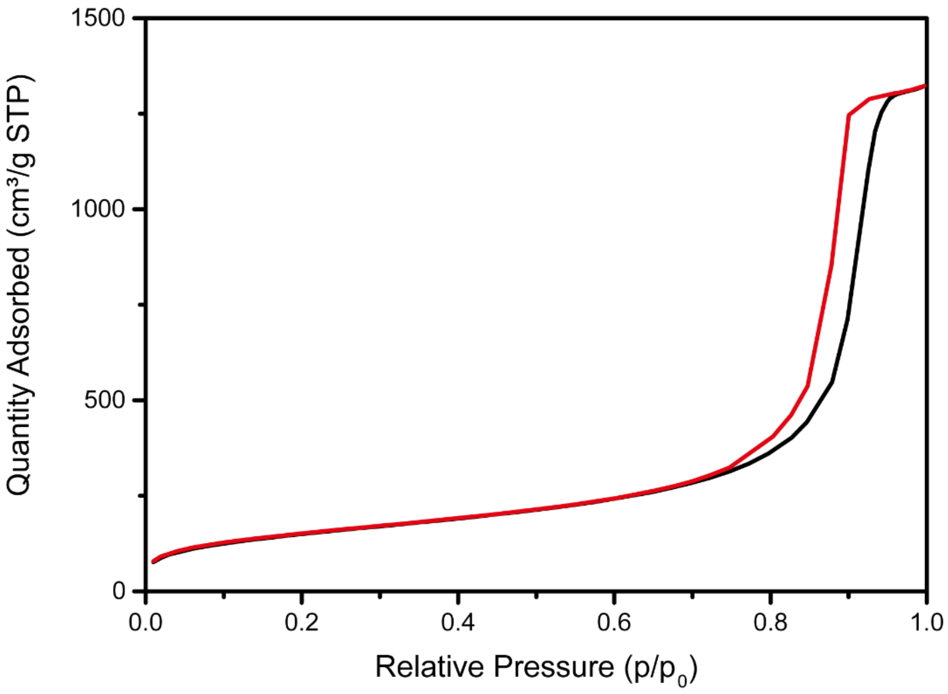


**Figure S13.** Type IV isotherm after $N_2$ physisorption, characterized by the closed hysteresis loop. Confirming the mesopores for this pristine catalyst material.

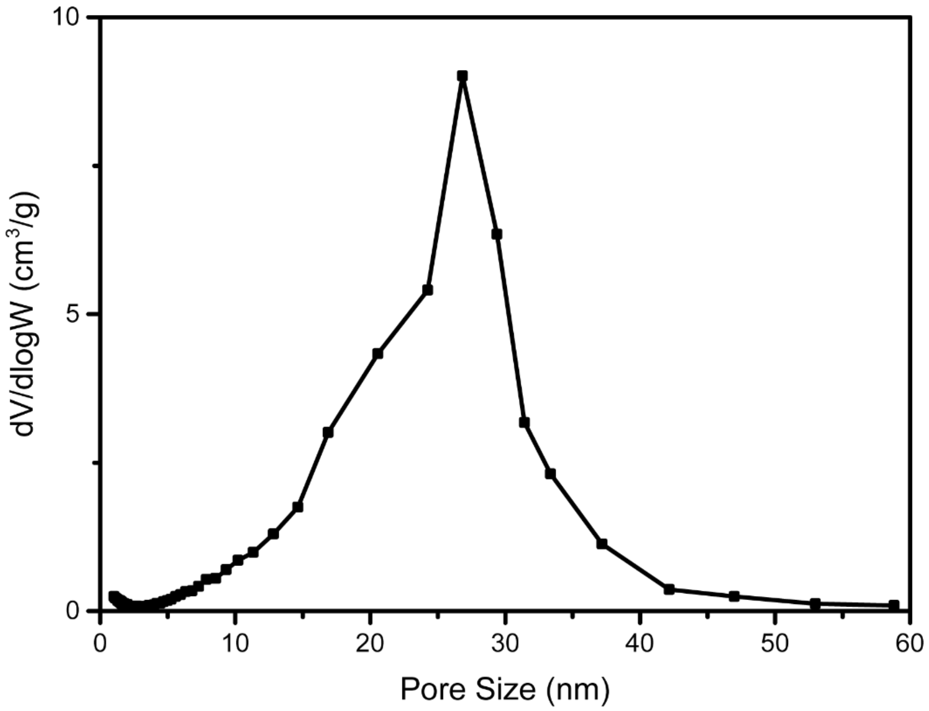


**Figure S14.** Pore Size Distribution obtained from the $N_2$ physisorption experiment, clearly showing the mesoporous nature of this catalyst material. No micropores and macropores are observed.

**Additional References**

[1] A.P. Hitchckock, *J. Electron Spectros. Relat. Phenomena,* **2015**, *200*, 49-63
[2] Y. Liu, F. Meirer, P. A. Williams, J. Wang, J. C. Andrews, P. Pianetta, *J. Synchrotron Radiat.*, **2012**, *19*, 281-287
